# Familial interstitial lung disease in four members of one family: a case series

**DOI:** 10.1186/1757-1626-2-9356

**Published:** 2009-12-19

**Authors:** Esam H Alhamad

**Affiliations:** 1 Department of Medicine, Division of Pulmonary Medicine, King Khalid University Hospital, King Saud University, Riyadh, 11415, Saudi Arabia

## Abstract

**Introduction:**

Familial interstitial lung disease has been reported worldwide, mostly in Europe and North America; limited information is available on the disease among Arab patients.

**Case presentation:**

A 45-year-old woman presented to our outpatient clinic with a 1-year history of progressive dyspnea. At the age of 37 years, based on clinical and radiological features, our patient was diagnosed with idiopathic pulmonary fibrosis. A family history showed that of five deceased siblings, four had died of disease of undetermined etiology. In addition, we screened other family members, and three were shown to have clinical, radiological, and pathological features consistent with interstitial pneumonia.

**Conclusion:**

Our report illustrates that younger age at presentation appears to be a common feature in patients with familial interstitial pneumonia. Otherwise, clinical, radiological, and histological features are indistinguishable from those of sporadic cases. Furthermore, our work highlights the importance of compiling a thorough family history in individuals presenting with cough and dyspnea, particularly in younger patients identified with idiopathic pulmonary fibrosis.

## Introduction

Familial idiopathic interstitial pneumonia is defined as when two or more members of the same family have clinical features of interstitial pneumonia [1,2]. To date, approximately 150 such families have been reported worldwide. Characteristically, patients with familial idiopathic pulmonary fibrosis (IPF) present at a younger age and screening of family members shows evidence of early interstitial changes in asymptomatic subjects [1,3,4]. Thus, examination of family members with idiopathic interstitial pneumonia provides an opportunity to uncover the pathogenesis of IPF at early stages when therapy might be effective. We describe four siblings with interstitial lung disease, three of whom were identified after taking a thorough family history from a patient, followed by family screening.

## Case presentation

### Case report 1

**Figure 1:**
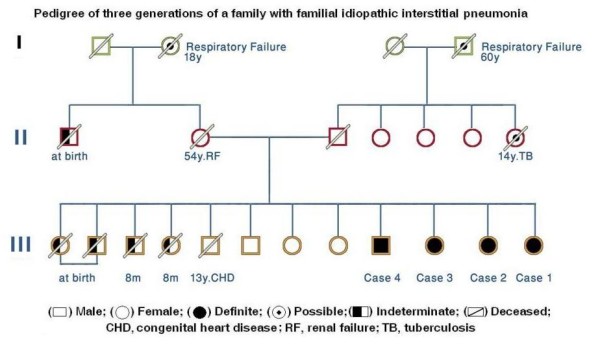
**Pedigree of three generations of a family with familial idiopathic interstitial pneumonia**.

A 45-year-old female Saudi Arabia, non-smoker presented to our outpatient clinic in January 2009 for a second opinion; she had a 1-year history of progressive dyspnea of New York Heart Association class III. The patient had been diagnosed with idiopathic pulmonary fibrosis (IPF) at the age of 37 years, based on a high-resolution computed tomography (HRCT) scan, and since that time she had taken corticosteroids, was home oxygen-dependent, and had been referred for lung transplantation abroad. Her initial presentation was shortness of breath, cough, and sputum production in the morning. She had worked as a schoolteacher for 15 years. A family history revealed that five siblings had died, one at the age of 13 years from congenital heart disease, and four from conditions of undetermined etiology, when all were less than 1 year old. In addition several members of her first and second generations had died from respiratory failure possibly related to underlying interstitial lung disease (Figure 1). Other family members revealed cough and dyspnea in cases 2 and 3, and cough with sputum production in case 4.

**Figure 2:**
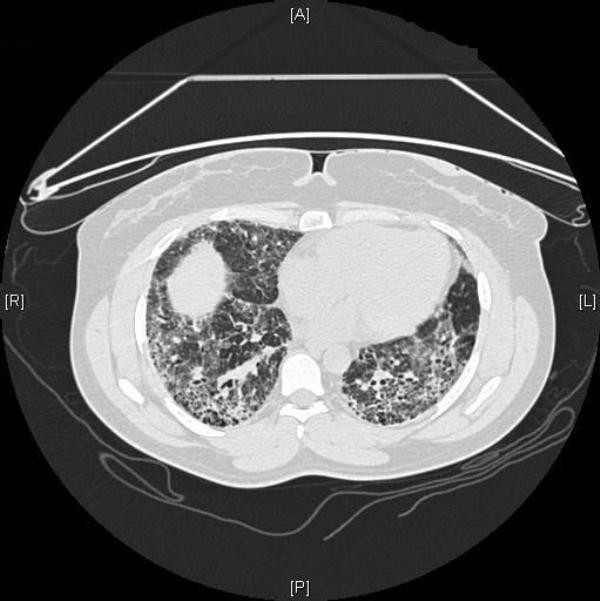
**Case 1**. High-resolution computed tomography (HRCT) scan at the basal portion of the lungs demonstrating ground-glass attenuation, reticular opacities, traction bronchiectasis, with honeycombing throughout both lungs.

On examination our patient had digital clubbing and bibasilar end-inspiratory crackles on auscultation. Pulmonary function and 6-minute walk test (6MWT) results are shown in Table 1. Pulmonary function studies and 6MWD data), which revealed a severe restrictive defect, with marked oxygen desaturation to 83% after the 6-minute walk even with 2 L of oxygen supplementation. Her Borg score was 6 at the end of the walk test indicating moderately severe dyspnea. A blood test was positive for antinuclear antibody (ANA) 1:640 of the speckled pattern, but other serological studies for collagen vascular disease were negative. A HRCT scan demonstrated ground glass attenuation, reticular opacities, traction bronchiectasis, and bronchiolectasis with honeycombing throughout both lungs (Figure 2). The patient was instructed to proceed with lung transplantation evaluation.

**Table 1:** Pulmonary function studies and 6MWD data.

Variables	Case 1	Case 2	Case 3	Case 4
FVC, % predicted	27.6	84.9	61.9	78

FEV1, % predicted	31	86.7	78.1	75

TLC, % predicted	31.9	70.6	43.7	87.6

DLCO, % predicted	-	30.2	32.9	82.4

PaO2, mmHg	68	68	87	-

PaCO2, mmHg	53	29	41	-

SaO2 at rest, %	93	94	97	-

Distance, m	122	372	362	506

Initial SPO2, %	93	97	95	96

Lowest SPO2, %	83	96	93	98

Initial Borg Score	0	0	0	0

Final Borg Score	6	1	3	1

### Case report 2

**Figure 3:**
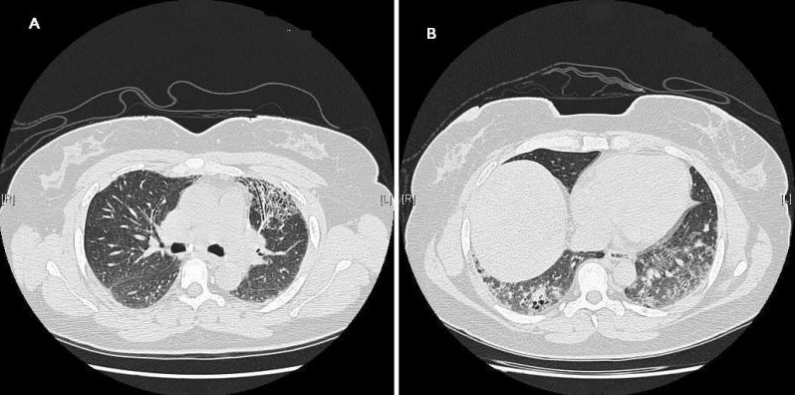
**Case 2**. (a) HRCT scan obtained at the upper lung zones shows reticular opacities and honeycombing. (b) HRCT scan from the basal parts of the lungs showing ground-glass attenuation, reticular and nodular densities, and traction bronchiectasis.

The 48-year-old female from Saudi Arabia, elder sister of case 1 complained of dyspnea on exertion, and cough with sputum production in the morning, for 3 years. She had been diagnosed with bronchial asthma at the age of 46 years. She had never smoked. She works as a schoolteacher. A physical review was unremarkable. On examination, she was not clubbed or cyanosed. Bibasilar end-inspiratory crackles were noted on auscultation. Pulmonary function test data as shown in Table 1. Pulmonary function studies and 6MWD data) indicated a restrictive defect with a markedly reduced diffusion capacity for carbon monoxide (DLCO). A blood test was positive for ANA 1:160, with a homogenous pattern. Other serological studies for collagen vascular disease were negative. A HRCT scan revealed inhomogeneous ground glass attenuation with reticular and nodular densities, associated with irregular pleural thickening with a shaggy pleuropulmonary interface, traction bronchiectasis, and fine honeycombing. HRCT scan obtained at the upper lung zones shows reticular opacities and honeycombing (Figure 3a). HRCT scan from the basal parts of the lungs showing ground-glass attenuation, reticular and nodular densities, and traction bronchiectasis (Figure 3b).

**Figure 4:**
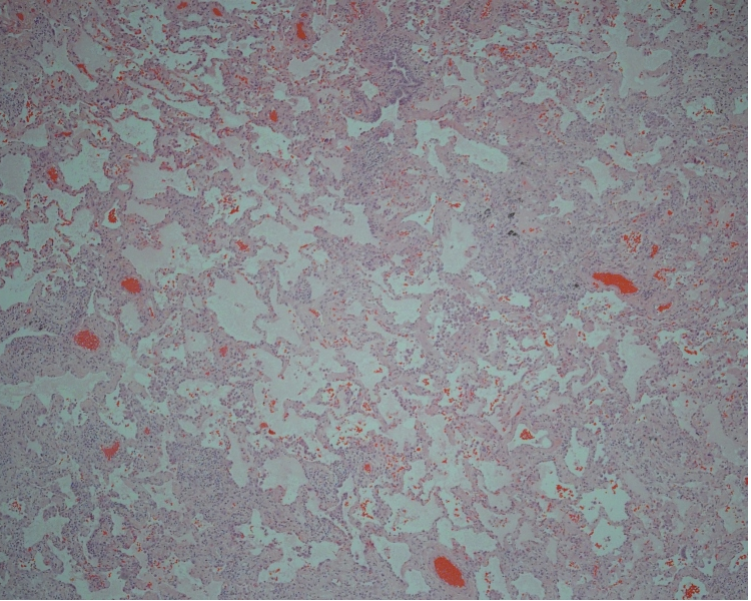
**Case 2**. Thoracoscopic lung biopsy showing areas of interstitial fibrosis alternating with less involved parenchyma. (haematoxylin and eosin stain) at ×10 magnification

A video-assisted thoracoscopic surgical lung biopsy was performed from the lingula, and the lateral basal segment of the left lower lobe showed an unclassifiable form of interstitial fibrosis (Figure 4). Thoracoscopic lung biopsy showing areas of interstitial fibrosis alternating with less involved parenchyma. (haematoxylin and eosin stain) at ×10 magnification). Our patient has clinically improved after commencement on prednisone, azathioprine, and N-acetylcysteine.

### Case report 3

**Figure 5:**
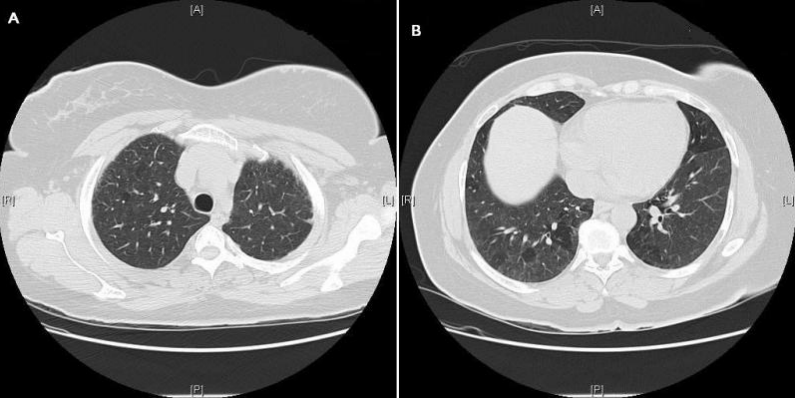
**Case 3**. (a) HRCT scan obtained from the upper lung zones shows interlobular reticular opacities and patchy ground-glass attenuation. (b) HRCT scan at the basal portion of the lungs showing multilobular regions of air trapping.

**Figure 6:**
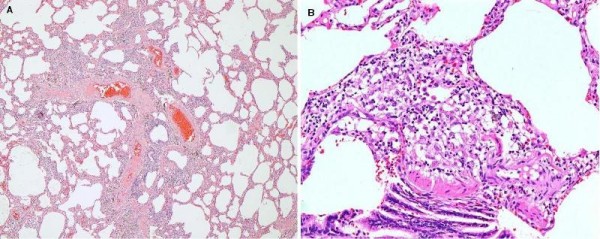
**Case 3**. (a) Thoracoscopic lung biopsy showing chronic interstitial inflammation with prominent bronchiolocentricity. (haematoxylin and eosin stain) at ×10 magnification. (b) Higher magnification showing poorly formed granuloma. (haematoxylin and eosin stain) at ×40 magnification

The third sister, 43 years of age from Saudi Arabia, reported dyspnea upon heavy exertion, an occasional dry cough, and chest discomfort for the past 1 year. Her medical history included diabetes mellitus and hypothyroidism, 6 years in duration. She had never smoked but had been exposed to secondhand smoke. She works as a schoolteacher. Physical examination did not reveal any abnormal finding. The results of physiologic testing are shown in Table 1. Pulmonary function studies and 6MWD data) and indicate a restrictive defect with a markedly reduced DLCO, but no significant oxygen desaturation after the 6MWT. Serological studies for collagen vascular disease were negative. HRCT revealed fine reticular infiltrates associated with focal ground glass attenuation in the upper zones, mild bronchial wall thickening, and multilobular areas of air trapping predominantly in the lower zones. HRCT scan obtained from the upper lung zones shows interlobular reticular opacities and patchy ground-glass attenuation (Figure 5a). HRCT scan at the basal portion of the lungs showing multilobular regions of air trapping (Figure 5b). A thoracoscopic lung biopsy demonstrated features consistent with hypersensitivity pneumonia. Thoracoscopic lung biopsy showing chronic interstitial inflammation with prominent bronchiolocentricity (haematoxylin and eosin stain) at ×10 magnification (Figure 6a). Higher magnification showing poorly formed granuloma. (haematoxylin and eosin stain) at ×40 magnification) (Figure 6b). The patient was urged to avoid passive smoking, and reported improvement in symptoms after commencing on prednisone and N-acetylcysteine.

### Case 4

**Figure 7:**
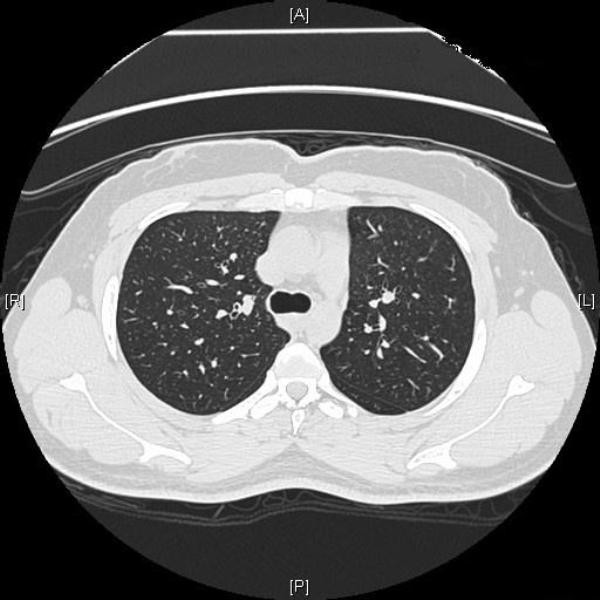
**Case 4**. HRCT scan obtained at the upper lung zones showing poorly defined centrilobular nodules with diffuse bronchial wall thickening throughout both lung fields.

**Figure 8:**
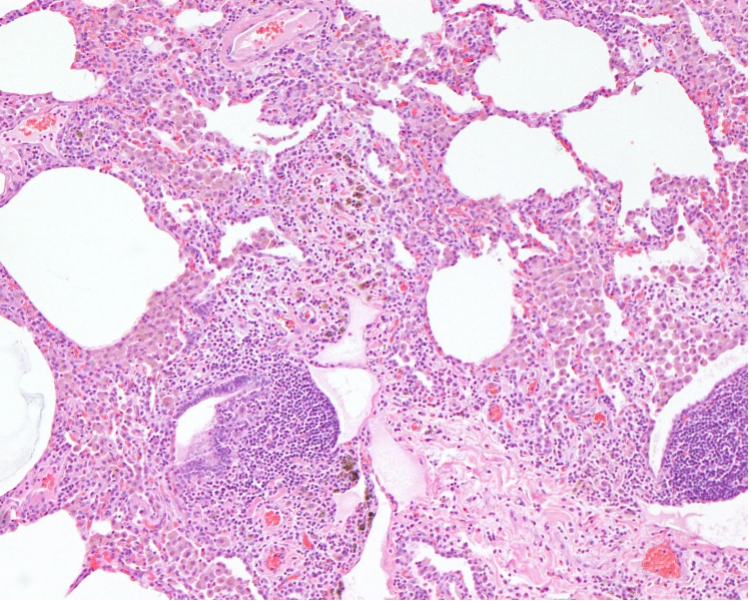
**Case 4**. Thoracoscopic lung biopsy showing pigmented macrophages in and around small bronchioles. (haematoxylin and eosin stain) at ×10 magnification

The younger brother, aged 30 years from Saudi Arabia, complained of sputum production and cough for the last 2 years. He had been diagnosed with bronchial asthma at the age of 28 years. He worked as a customer service representative, with frequent exposure to diesel exhaust. He had smoked 20-30 cigarettes daily for 10 years. On examination, he was not clubbed or cyanosed, and his chest was clear on auscultation. The pulmonary function test demonstrated a mild obstructive and restrictive defect with no significant impairment in DLCO and a normal 6MWT Table 1. Pulmonary function studies and 6MWD data). HRCT showed diffuse bronchial wall thickening and poorly defined centrilobular nodules (Figure 7). HRCT scan obtained at the upper lung zones showing poorly defined centrilobular nodules with diffuse bronchial wall thickening throughout both lung fields). A surgical lung biopsy demonstrated pigmented macrophages in and around the small bronchioles (Figure 8). Thoracoscopic lung biopsy showing pigmented macrophages in and around small bronchioles, interstitial thickening and peribronchiolar inflammatory changes (haematoxylin and eosin stain) at ×10 magnification). This appearance, in line with clinical and radiological findings, was consistent with the diagnosis of respiratory bronchiolitis-associated interstitial lung disease (RB-ILD). The patient quit smoking after counseling.

## Discussion

Idiopathic interstitial pneumonias are a group of diffuse infiltrative lung diseases of unknown etiology composed of several clinicopathologic subtypes including IPF also known as usual interstitial pneumonia (UIP), which is the most common subtype; nonspecific interstitial pneumonia (NSIP); respiratory bronchiolitis-associated interstitial lung disease (RB-ILD); desquamative interstitial pneumonia (DIP); cryptogenic organizing pneumonia (COP); acute interstitial pneumonia (AIP); and lymphocytic interstitial pneumonia (LIP) [5]. Familial IPF is defined as when two or more members of the same family show clinical features of idiopathic interstitial pneumonia [1,2]. The condition was first described in 1907 by Sandoz [6]. Another report [7] described IPF in identical twin sisters and since that time 15 definite cases and 3 other probable cases of pulmonary fibrosis have been diagnosed in the same family [8]. The exact prevalence of familial IPF is unknown, however two recent studies suggest that 0.5-3.7% of all patients with IPF have disease of the familial type [9,10]. The mode of inheritance of familial IPF is not entirely clear; however, the observed father-to-son transmission excludes X-chromosome linkage, whereas a single report suggested that inheritance is an autosomal recessive trait [11]. The majority of reported cases demonstrate an autosomal dominant characteristic with reduced penetrance [1,2,8,10,12]. Sporadic IPF usually presents between 50 and 70 years of age. Progressive dyspnea upon exertion, nonproductive cough, clubbing of the digits, and bibasilar end-inspiratory crackles constitute the typical clinical presentation. In a retrospective study, Lee and colleagues examined 27 patients from 15 families with familial IPF [2]. The clinical findings, pulmonary function testing, pathologic changes, radiographic features, and survival data were similar in patients with familial IPF and non-familial IPF. HRCT scans were performed on 66% of patients and bilateral irregular linear opacities were seen in all cases. Other findings included a subpleural predominance of linear opacities (94%), subpleural traction bronchiectasis (82%), emphysematous changes (18%), ground glass opacities (12%), and bronchial wall thickening with irregularities (6%). Although the cited investigators concluded that the radiographic features of familial IPF and non-familial IPF were similar, a lower incidence of honeycombing (29%) was seen in familial IPF patients compared to those with non-familial IPF. Nishiyama and colleagues [13] described HRCT findings in nine patients with biopsy-proven familial IPF, and found a resemblance between familial IPF and non-familial IPF, except that the prevalence of honeycombing (33%) and lower lung zone distribution (67%) was lower in familial IPF patients compared to those with non-familial IPF. Nevertheless, a follow-up HRCT study revealed disease progression and honeycomb development in the majority of patients with familial IPF [2,13]. Age at onset appears to be lower in patients with familial IPF, who usually present with symptoms during the third or fourth decade of life [14]. This probably results from early screening of non-affected family members as demonstrated in our present case report. Interestingly, 50% of asymptomatic first-degree relatives of subjects affected by familial IPF have a pattern of alveolar inflammation similar to that observed in patients with sporadic IPF in the absence of detectable abnormalities in pulmonary function or chest radiography [4]. In another study, Steel and associates reported on the clinical features of familial idiopathic interstitial pneumonia, and found that almost 8% of subjects without symptoms of pulmonary fibrosis had HRCT findings consistent with probable or definite interstitial pneumonia [1]. Rosas and colleagues evaluated family members of patients affected by familial IPF to identify asymptomatic subjects with interstitial lung disease (ILD) [3]. The cited authors found that 22% of asymptomatic subjects showed radiographic evidence of ILD when screened by HRCT. A consistent finding in previous studies was that a history of cigarette smoking was strongly associated with the development of early ILD in family members of those with familial interstitial pneumonia [1,3]. This suggests that a genetically susceptible individual may develop lung injury from cigarette smoking and exposure to other environmental toxins that may stimulate fibroproliferation, further increasing the risk of developing ILD and progression of pulmonary fibrosis. Several studies have revealed heterogeneity of histopathologic findings in family members of individuals with familial IPF [1,3,15]. In one study, approximately 45% of family members showed more than one histologic subtype when relatives of those affected by familial interstitial pneumonia were surveyed [1]. The reported combinations of histologic types within families include: UIP and NSIP; UIP, NSIP, and COP; UIP, NSIP, and RB-ILD; UIP and unclassified ILD; or UIP and hypersensitivity pneumonia [1,3,15]. Although the clinical and morphologic features of various histologic types of idiopathic interstitial pneumonia differ from those of UIP, the conditions may perhaps represent gradations of a spectrum of which UIP is the final histopathologic result. Such speculation is supported by the simultaneous occurrence of UIP and NSIP in the same patient with idiopathic interstitial pneumonia [16], as well the similarity in gene expression profile between UIP and NSIP [17] patients. In the present case report, an examination of the pedigree of three generations revealed several deaths that occurred both during infancy and adult life. It is possible to speculate that affected individuals might share a single candidate genetic alteration such as a mutation in the surfactant protein C (SP-C) gene (SFTPC), resulting in development of pulmonary fibrosis in our studied family. However, other investigators believe that early onset cases of ILD are distinct, arising from a pathogenic mechanism different from that causing adult onset occurrence of familial IPF [10]. Four surfactant proteins (SPs) have been identified and are divided into two groups: the hydrophilic proteins SP-A and SP-D and the hydrophobic proteins SP-B and SP-C. SP-A, SP-B, and SP-D are synthesized by both alveolar cells and nonciliated bronchiolar cells of the lung, whereas SP-C is produced only by alveolar type II cells. SP-C plays a critical role in reducing surface tension in the alveoli, whereas SP-C absence or mutation in SFTPC mutant patients causes mechanical injury to the respiratory epithelium leading to respiratory failure and severe ILD [18]. This was first reported by Nogee and colleagues who described a mother with desquamative interstitial pneumonia, and her infant with NSIP [19]. In another study, Thomas and co-workers reported a heterozygous T to A substitution in the fifth exon of the SFTPC gene that was associated with adult onset UIP with childhood cellular NSIP [15]. Recently, Selman and colleagues evaluated genetic polymorphic variants of the surfactant proteins SP-A1, SP-A2, SP-B, SP-C, and SP-D in a group of patients with IPF [20]. The SP-A1_6A4 allele was noted to be associated with nonsmoker IPF, whereas the SP-B B1580_C allele was more associated with smoker IPF patients compared to control subjects. Thus, in line with previous studies, SPs appear to play an essential role in the pathogenesis of both familial and sporadic IPF. Pulmonary fibrosis has also been associated with other rare genetic disorders yielding pleiotropic presentations, including Hermansky-Pudlak syndrome, neurofibromatosis, tuberous sclerosis, Niemann-Pick disease, Guacher disease, familial hypocalciuric hypercalcemia, and congenital dyskeratosis. The management of patients with familial IIP is similar to that applied to sporadic cases and is dependent upon the type of IIP diagnosed, However, to date, lung transplantation is the only therapy that has been shown to improve survival in younger individuals with pulmonary fibrosis.

## Conclusion

Our report illustrates the importance of obtaining a detailed family history, as such information can assist in the diagnosis and early intervention in ILD patients. Clinical findings, histopathology, and radiographic features are indistinguishable between familial idiopathic interstitial pneumonia and sporadic cases. Younger age at diagnosis is a common finding in patients with the former condition. Cigarette smoking is strongly associated with development of early ILD in family members with familial interstitial pneumonia, and both patients and asymptomatic individuals should be urged to stop smoking.

## Abbreviations

6MWD: six-minute walk distance in meters; FVC: forced vital capacity; FEV_1_: forced vital capacity in 1 second; PaO_2_: partial pressure of oxygen; PaCO_2_: partial pressure of carbon dioxide; SaO_2_: oxygen saturation; SPO_2_: oxygen saturation by pulse oximetry; TLC: total lung capacity.

## Consent

Written informed consent was obtained from all patients for publication of this case report and the accompanying images. A copy of the written consent is available for review by the Editor-in-Chief of the Journal.

## Competing interests

The author declares that they have no competing interests.

## References

[B1] Steele MP Speer MC Loyd JE Brown KK Herron A Slifer SH Burch LH Wahidi MM Phillips JA Sporn TA McAdams HP Schwarz MI Schwartz DA Clinical and pathologic features of familial interstitial pneumonia Am J Respir Crit Care Med 2005 172 9 1146 1152 10.1164/rccm.200408-1104OC 16109978 PMC2718398

[B2] Lee HL Ryu JH Wittmer MH Hartman TE Lymp JF Tazelaar HD Limper AH Familial idiopathic pulmonary fibrosis: clinical features and outcome Chest 2005 127 6 2034 2041 10.1378/chest.127.6.2034 15947317

[B3] Rosas IO Ren P Avila NA Chow CK Franks TJ Travis WD McCoy JP Jr May RM Wu HP Nguyen DM Arcos-Burgos M MacDonald SD Gochuico BR Early interstitial lung disease in familial pulmonary fibrosis Am J Respir Crit Care Med 2007 176 7 698 705 10.1164/rccm.200702-254OC 17641157 PMC1994234

[B4] Bitterman PB Rennard SI Keogh BA Wewers MD Adelberg S Crystal RG Familial idiopathic pulmonary fibrosis. Evidence of lung inflammation in unaffected family members N Engl J Med 1986 314 21 1343 1347 3702942 10.1056/NEJM198605223142103

[B5] American Thoracic Society/European Respiratory Society International Multidisciplinary Consensus Classification of the Idiopathic Interstitial Pneumonias. This joint statement of the American Thoracic Society (ATS), and the European Respiratory Society (ERS) was adopted by the ATS board of directors, June 2001 and by the ERS Executive Committee, June 2001 Am J Respir Crit Care Med 2002 165 2 277 304 11790668 10.1164/ajrccm.165.2.ats01

[B6] Sandoz E Uber zwei Falle von fotaler Bronchektasie Beitr Pathol Anat 1907 41 495 516

[B7] Peabody JW Peabody JW Jr Hayes EW Hayes EW Jr Idiopathic pulmonary fibrosis; its occurrence in identical twin sisters Dis Chest 1950 18 4 330 344 10.1378/chest.18.4.330 14778377

[B8] Marney A Lane KB Phillips JA Riley DJ Loyd JE Idiopathic pulmonary fibrosis can be an autosomal dominant trait in some families Chest 2001 120 Suppl 1 56S 10.1378/chest.120.1_suppl.S56 11451917

[B9] Hodgson U Laitinen T Tukiainen P Nationwide prevalence of sporadic and familial idiopathic pulmonary fibrosis: evidence of founder effect among multiplex families in Finland Thorax 2002 57 4 338 342 10.1136/thorax.57.4.338 11923553 PMC1746288

[B10] Marshall RP Puddicombe A Cookson WO Laurent GJ Adult familial cryptogenic fibrosing alveolitis in the United Kingdom Thorax 2000 55 2 143 146 10.1136/thorax.55.2.143 10639533 PMC1745672

[B11] Tsukahara MKT Interstitial pulmonary fibrosis in two sisters. Possible autosomal recessive inheritance Jinrui Idengaku Zasshi 1983 28 2 263 267 6678314 10.1007/BF01876789

[B12] Musk AW Zilko PJ Manners P Kay PH Kamboh MI Genetic studies in familial fibrosing alveolitis. Possible linkage with immunoglobulin allotypes (Gm) Chest 1986 89 2 206 210 10.1378/chest.89.2.206 3484694

[B13] Nishiyama O Taniguchi H Kondoh Y Kimura T Katoh T Oishi T Matsumoto S Yokoi T Takagi K Shimokata K Johkoh T Muller NL Familial idiopathic pulmonary fibrosis: serial high-resolution computed tomography findings in 9 patients J Comput Assist Tomogr 2004 28 4 443 448 10.1097/00004728-200407000-00002 15232373

[B14] Barzo P Familial idiopathic fibrosing alveolitis Eur J Respir Dis 1985 66 5 350 352 4018187

[B15] Thomas AQ Lane K Phillips J Prince M Markin C Speer M Schwartz DA Gaddipati R Marney A Johnson J Roberts R Haines J Stahlman M Loyd JE Heterozygosity for a surfactant protein C gene mutation associated with usual interstitial pneumonitis and cellular nonspecific interstitial pneumonitis in one kindred Am J Respir Crit Care Med 2002 165 9 1322 1328 10.1164/rccm.200112-123OC 11991887

[B16] Flaherty KR Travis WD Colby TV Toews GB Kazerooni EA Gross BH Jain A Strawderman RL Flint A Lynch JP Martinez FJ Histopathologic variability in usual and nonspecific interstitial pneumonias Am J Respir Crit Care Med 2001 164 9 1722 1727 11719316 10.1164/ajrccm.164.9.2103074

[B17] Yang IV Burch LH Steele MP Savov JD Hollingsworth JW McElvania-Tekippe E Berman KG Speer MC Sporn TA Brown KK Schwarz MI Schwartz DA Gene expression profiling of familial and sporadic interstitial pneumonia Am J Respir Crit Care Med 2007 175 1 45 54 10.1164/rccm.200601-062OC 16998095 PMC1899261

[B18] Whitsett JA Genetic basis of familial interstitial lung disease: misfolding or function of surfactant protein C? Am J Respir Crit Care Med 2002 165 9 1201 1202 10.1164/rccm.2203017 11991863

[B19] Nogee LM Dunbar AE Wert SE Askin F Hamvas A Whitsett JA A mutation in the surfactant protein C gene associated with familial interstitial lung disease N Engl J Med 2001 344 8 573 579 10.1056/NEJM200102223440805 11207353

[B20] Selman M Lin HM Montaño M Jenkins AL Estrada A Lin Z Wang G DiAngelo SL Guo X Umstead TM Lang CM Pardo A Phelps DS Floros J Surfactant protein A and B genetic variants predispose to idiopathic pulmonary fibrosis Hum Genet 2003 113 6 542 550 10.1007/s00439-003-1015-4 13680361

